# Acute Effects of Outdoor Air Pollution on Emergency Department Visits Due to Five Clinical Subtypes of Coronary Heart Diseases in Shanghai, China

**DOI:** 10.2188/jea.JE20140044

**Published:** 2014-11-05

**Authors:** Juan Xie, Mingzhen He, Weiying Zhu

**Affiliations:** 1 The Trauma Emergency & Critical Care Medicine Center, the Fifth People’s Hospital of Shanghai, Fudan University, Shanghai, China; 2 Changzhou Center for Disease Control and Prevention, Changzhou, Jiangsu Province, China

**Keywords:** air pollution, coronary heart disease, clinical subtypes, emergency department visits

## Abstract

**Background:**

Air pollution can be a contributing cause to the development and exacerbation of coronary heart disease (CHD), but there is little knowledge about the acute effects of air pollution on different clinical subtypes of CHD.

**Methods:**

We conducted a time-series study to investigate the association of air pollution (particulate matter with an aerodynamic diameter < 10 µm [PM_10_], sulfur dioxide [SO_2_], and nitrogen dioxide [NO_2_]) on emergency department (ED) visits due to five different subtypes of CHD in Shanghai, China, from 2010 to 2012. We applied an over-dispersed Poisson generalized addictive model to analyze the associations after controlling for the seasonality, day of the week, and weather conditions.

**Results:**

We identified a total of 47 523 ED visits for CHD. A 10-µg/m^3^ increase in the present-day concentrations of PM_10_, SO_2_, and NO_2_ was associated with respective increases of 1.10% (95% confidence interval [CI] 0.33%–1.87%), 0.90% (95% CI −0.14%–1.93%), and 1.44% (95% CI 0.63%–2.26%) for total ED visits for CHD. These associations varied greatly by clinical type, with strong effects on sudden cardiac death, moderate effects on acute myocardial infarction and angina, weak effects on ischemic cardiomyopathy, and no effect on occult CHD. The associations were stronger among people aged 65 years or more than in younger individuals and in the cool season versus the warm one.

**Conclusions:**

Outdoor air pollution may have different effects of air pollution on 5 subtypes of CHD. Our results might be useful for the primary prevention of various subtypes of CHD exacerbated by air pollution.

## INTRODUCTION

Coronary heart disease (CHD), also known as coronary artery disease or ischemic heart disease, is one of the leading causes of deaths and hospital admissions worldwide. It constitutes the leading cause of disease burden in the world according to the 2010 Global Burden of Disease Study (GBD).^1^^,^^2^ Traditional risk factors have been well documented, including aging, male sex, family history of CHD, smoking, physical inactivity, hypertension, high blood cholesterol, diabetes mellitus, obesity, unhealthy diet, and high stress.^3^^,^^4^ In recent years, toxicological and epidemiological studies have provided increasing evidence that ambient air pollution likely contributes to the development and exacerbation of CHD.^5^^,^^6^ Unlike traditional risk factors, exposure to air pollution is almost ubiquitous and thus may constitute a high burden of disease that should arouse substantial public health concerns, especially in developing countries where air pollution levels are high, such as China. For example, the 2010 GBD estimated that ambient fine particulate matter (PM) air pollution causes 280 000 CHD deaths annually in China.^1^

CHD can be clinically classified into 5 subtypes: occult CHD, angina, acute myocardial infarction (AMI), ischemic cardiomyopathy (ICM), and sudden cardiac death (SCD). Most previous epidemiological studies linking short-term exposure to air pollution and CHD did not focus on specific types of CHD, or only limited findings to AMI and SCD. There is little knowledge about the possible associations of air pollution and occult CHD, angina, and ICM.

Therefore, we performed a time-series study to investigate the acute effects of outdoor air pollution on emergency department (ED) visits due to five different clinical types of CHD in Shanghai, China.

## METHODS

### Health data

The daily ED visit data for CHD were obtained from the Fifth People’s Hospital of Shanghai, Fudan University during the period of 2010 to 2012 (1096 days). As the largest hospital in Minhang District, it serves more than 2 million residents in and around this district, which has a residential area of almost 300 square kilometers. In recent years, it has had more than 65 000 ED visits annually. For each ED visit, the physician must complete a medical record including symptoms, body examination, primary diagnosis, treatment, and basic personal information (admitted time, name, age, sex, address). All medical records were entered into the electronic health record computer system. The specific subtypes of CHD were determined by physicians according to patients’ symptoms, inquiries, complaints, and findings on medical inspection, such as electrocardiogram (ECG) and serum cardiac biomarker analysis.

For this study, we retrieved all ED records diagnosed as CHD from the database. First, we excluded the patients who resided outside the district (about 4%) according to the recorded addresses. Then, five cardiologists who were blinded to air quality data reviewed the electronic files and validated the diagnosis of each specific type of CHD. If the subtype diagnosis was not available in the records, it was re-ascertained according to the patient’s major complaints and overwhelming abnormality (ie, primary cause of ED visits) in the body examinations. We further excluded any patients whose records did not support the diagnosis of a specific subtype. At last, we summarized daily number of ED visits for each specific subtype of CHD.

The major criteria for identifying the subtypes of CHD were as follows:

• Occult CHD: ECG shows ST-segment depression, and low or inverted T waves, but there is absence of obvious and typical cardiac symptoms.^7^
• Angina: The presence of substernal chest pain; discomfort that is provoked by physical exertion or emotional stress; chest pain that can be relieved by rest and/or nitroglycerin. Chest pain was defined as atypical angina if at least two of the above three criteria were present.^8^
• AMI: Clinical history of ischemic-type chest pain lasting for more than 20 minutes; changes in serial ECG tracings, typically including pathological Q waves or ST elevation; dynamic changes in levels of serum cardiac biomarkers, typically a rise in troponin and creatine kinase isoenzyme MB.^9^
• ICM: A history of AMI or revascularization, such as coronary artery bypass grafting and percutaneous coronary intervention; >75% stenosis of left main or proximal left anterior descending coronary artery; >75% stenosis of two or more epicardial vessels. Its clinical manifestation can be cardiac enlargement, heart failure, and arrhythmia.^10^
• SCD: The absence of a pulse and death within 1 hour after the onset of acute cardiac symptoms.^11^

### Environmental data

Daily 24-h average concentrations of PM_10_ (particulate matter with an aerodynamic diameter less than 10 µm), sulfur dioxide (SO_2_), and nitrogen dioxide (NO_2_) were obtained from a fixed-site air quality monitoring station less than 1 km away from the hospital. The location of the station was not in the direct vicinity of traffic or of industrial air pollution sources. Methods based on tapered element oscillating microbalance, ultraviolet fluorescence, and chemiluminescence were used for the measurement of PM_10_, SO_2_, and NO_2_, respectively.

Daily mean temperature and relative humidity were obtained from the database of the Shanghai Meteorological Bureau in order to allow for adjustment for the potential confounding effects of weather conditions on CHD.

### Ethics

This study does not involve experimental animals. Informed consent was waived for this study because all health data were analyzed at the aggregate level, no individual records/information for patients were involved, and no patients were contacted. The protocol of this study was approved by the Institutional Review Board of the Fifth People’s Hospital of Shanghai, Fudan University.

### Statistical analysis

A time-series design was used to investigate the acute effects of short-term exposure to air pollution on ED visits for CHD.^12^ This approach can automatically control for time-invariant confounders (such as age, sex, family history, and socioeconomic characteristics) by examining the same population repeatedly over time. The time-series design can also adjust for time-varying confounders by incorporating a smooth function of the unmeasured time trends due to physical activity level, diet structure, and smoking, as well as blood pressure, glucose, and lipid levels. Time-series models have become a standard analytic method when exploring the acute health effects of air pollution and temperature variations based on aggregate data.^13^

For the time-series design, we applied a generalized additive model (GAM) to analyze the data. Because daily ED visits typically followed an over-dispersed Poisson distribution, we used quasi-Poisson regression in the GAM.^14^ Consistent with previous time-series studies,^15^ we empirically incorporated several covariates in the GAM to control potential confounding effects: (1) a natural cubic smooth function of calendar time with 7 degrees of freedom (df) per year, to exclude unmeasured long-term and seasonal trends longer than 2 months; (2) separate natural smooth functions of the current-day (lag 0) and 3-day moving average (lag 1–3) for mean temperature (6 df) and relative humidity (3 df) to control for the potential nonlinear confounding effects of weather conditions; (3) an indicator variable for “day of the week,” to adjust for the day-in-week variation of the ED visits; and (4) an indicator variable for holidays. After the basic model was built, we introduced a priori in turn each air pollutant’s concentrations on the concurrent day (lag 0), because previous studies indicated that current-day air pollution was most closely correlated with cardiovascular events.^16^ We also evaluated the effects of air pollution on the previous day (lag 1) and previous 2 day (lag 2).

Because the morbidity of CHD and the exposure to air pollution may have varied by age, sex, and season, we explored the modifying patterns of these variables on air pollution-related effects on CHD by stratified analyses. We did not analyze the age-, sex-, and season-specified effects on the five subtypes of CHD because of the limited number of daily ED visits.

We flexibly plotted the concentration-response relationships for air pollutants and ED visits for CHD using 3 df for the spline function and conducted four sensitivity analyses to check the stability of our results. First, we fitted two-pollutant models after controlling for other pollutants in turn. Second, given that it was not easy to determine df for the smoothness of time, we selected alternative df of 4–10 per year. Third, we examined the association between air pollution and ED visits for injuries that were not biologically related to air pollution using the same basic model. Fourth, we performed additional analyses using the time-stratified case-crossover method, an alternative study design for time-series models.^17^

Statistical tests were two-sided, and effects of *P* < 0.05 were considered statistically significant. All models were fitted using R software version 2.15.3 (R Foundation for Statistical Computing, Vienna, Austria) with time-series analyses using the “mgcv” package, and case-crossover analyses using the “season” package. The results were presented as the percentage and 95% confidence intervals (CIs) of change in number of daily ED visits per 10 µg/m^3^ increase in pollutant concentrations.

## RESULTS

Table 1 summarizes the descriptive statistics in this study. During the study period of 2010–2012 (1096 days), we identified a total of 51 665 ED visits for CHD, of which 92% were classifiable according to subtype. On average, occult CHD, angina, AMI, ICM, and SCD accounted for 32%, 18%, 12%, 30%, and 5% of the total CHD-related ED visits, respectively. As shown in eFigure 1 in the supplementary materials, the number of daily ED visits for CHD followed an apparent seasonal pattern, with a peak in winter. The winter-peak pattern was most evident for AMI and SCD; there were slightly more ED visits for angina and ICM in winter than the other subtypes, and there was no appreciable seasonal trend for occult CHD. There were more ED visits among males and people over age 65 years. The annual mean 24 h-average concentrations were 79 µg/m^3^ for PM_10_, 30 µg/m^3^ for SO_2_, and 56 µg/m^3^ for NO_2_. The PM_10_ concentrations in Shanghai were almost on a par with the national average level in China, but much higher than those measured in developed countries. All three pollutants had apparent seasonal trends, with a peak in winter (See eFigure 2, eFigure 3, and eFigure 4). The annual average temperature and humidity were 17°C and 68%, respectively. Generally, the Spearman correlation among PM_10_, SO_2_, and NO_2_ were moderate (coefficients ranging from 0.66 to 0.71), and were inverse and weak with temperature (ranging from 0.24 to 0.40) and relative humidity (ranging from 0.19 to 0.46).

**Table 1.** Descriptive statistics on daily emergency room visits for CHD, air pollution, and weather conditions in this study in Shanghai, China, from 2010–2012

	Mean	SD	Minimum	Median	IQR	Maximum
Emergency room visits for CHD	36	12	16	34	24	102
Occult CHD	14	4	7	13	9	36
Angina	8	2	3	7	5	20
Acute myocardial infarction	5	1	1	4	2	13
Ischemic cardiomyopathy	13	4	6	13	9	36
Sudden cardiac death	3	1	0	2	2	7
Air pollutant concentration (µg/m^3^)						
PM_10_	79	61	7	64	52	600
SO_2_	30	17	6	26	24	134
NO_2_	56	21	16	54	27	152
Weather conditions						
Mean temperature (°C)	17	9	−2	17	15	36
Mean relative humidity (%)	68	12	23	23	68	95

CHD, coronary heart disease; IQR, interquartile range; SD, standard deviation; PM_10_, particulate matter with an aerodynamic diameter less than 10 µm; SO_2_, sulfur dioxide; NO_2_, nitrogen dioxide.

The short-term associations of air pollutants with ED visits for CHD and each clinical type of CHD are described in Table 2. A steady trend with smaller effect estimates was detected using longer lag days. Findings for single-day lags supported the notion that the a priori lag (lag 0) provided the largest effect estimates, whereas the effect estimates decreased substantially and even became insignificant using lag 2 and lag 3. Significant effects were observed with lag 0 or 1 for PM_10_ and NO_2_, but not for SO_2_. For example, 10-µg/m^3^ increases in the present-day concentrations of PM_10_, SO_2_, and NO_2_ were associated with increases of 1.10% (95% CI 0.33%–1.87%), 0.90% (95% CI −0.14%–1.93%), and 1.44% (95% CI 0.63%–2.26%), respectively, in total number ED visits for CHD. For the specific type of CHD, the strongest effects were found for SCD, followed by AMI and angina. Air pollution was weakly associated with ICM, but not significantly associated with occult CHD.

**Table 2.** Percent increase (mean and 95% confidence intervals) in daily emergency room visits for CHD associated with a 10-µg/m^3^ increase in pollutant concentrations on the concurrent day in Shanghai, China, from 2010–2012

Emergency room visits	Lag days	PM_10_	SO_2_	NO_2_
Total CHD	0	1.10 (0.33, 1.87)	0.90 (−0.14, 1.93)	1.44 (0.63, 2.26)
	1	0.54 (−0.25, 1.33)	0.87 (−0.18, 1.91)	1.20 (0.38, 2.02)
	2	−0.58 (−1.39, 0.23)	0.63 (−0.41, 1.67)	0.67 (−0.15, 1.48)
Occult CHD	0	0.30 (−0.86, 1.46)	−0.40 (−1.04, 0.25)	0.24 (−0.67, 1.16)
	1	0.04 (−1.18, 1.26)	−0.47 (−1.26, 0.33)	−0.70 (−1.69, 0.30)
	2	−0.28 (−1.48, 0.92)	0.13 (−0.67, 0.93)	−0.67 (−1.60, 0.26)
Angina	0	1.50 (0.65, 2.34)	0.90 (−0.20, 1.99)	1.94 (0.73, 3.15)
	1	0.71 (−0.08, 1.50)	0.67 (−0.38, 1.71)	0.98 (−0.23, 2.20)
	2	0.58 (−0.37, 1.53)	−0.53 (−2.10, 1.04)	0.17 (−0.98, 1.32)
Acute myocardial infarction	0	2.30 (1.41, 3.18)	1.40 (0.36, 2.43)	3.44 (2.23, 4.65)
	1	0.84 (−0.05, 1.73)	0.92 (−0.13, 1.96)	1.60 (0.39, 2.81)
	2	−0.23 (−1.04, 0.58)	−0.43 (−1.47, 0.61)	0.57 (−0.83, 1.97)
Ischemic cardiomyopathy	0	0.80 (0.22, 1.37)	0.90 (−0.54, 2.33)	1.04 (−0.01, 2.09)
	1	0.55 (−0.16, 1.26)	0.57 (−1.09, 2.22)	0.60 (−0.36, 1.56)
	2	0.18 (−0.63, 0.99)	−0.43 (−2.00, 1.14)	0.07 (−0.78, 0.92)
Sudden cardiac death	0	3.10 (2.13, 4.06)	1.30 (0.10, 2.49)	4.24 (2.68, 5.80)
	1	1.20 (0.41, 1.99)	0.18 (−0.78, 1.14)	1.80 (0.31, 3.28)
	2	0.18 (−0.78, 1.14)	0.53 (−0.59, 1.65)	0.67 (−0.62, 1.95)

CHD, coronary heart disease; PM_10_, particulate matter with an aerodynamic diameter less than 10 µm; SO_2_, sulfur dioxide; NO_2_, nitrogen dioxide.

Table 3 presents the effect estimates on ED visits for total CHD classified by age group, sex, and season. We found significantly larger effects for people aged 65 years or more than those less than 65 years of old. The between-sex results were similar, with a slightly larger effect among males. As for the season-stratified analysis, the effect estimates were appreciably larger during the cool period. In the warm period, we failed to detect significant effects. Interestingly, SO_2_ was weakly associated with CHD among old people ≥65 years and in the cool period.

**Table 3.** Age-, gender-, and season-specific percent increase (mean and 95% confidence intervals) in emergency room visits for coronary heart disease associated with a 10-µg/m^3^ increase in pollutant concentrations on the concurrent day in Shanghai, China, from 2010–2012

	PM_10_	SO_2_	NO_2_
Age group			
<65	0.60 (−0.37, 1.56)	0.20 (−0.84, 0.45)	−0.13 (1.15, 0.88)
≥65	2.97 (2.14, 3.80)	0.37 (0.04, 0.70)	4.13 (2.92, 5.34)
Sex			
Male	1.30 (0.53, 2.07)	1.20 (−0.04, 2.43)	1.94 (1.05, 2.84)
Female	0.90 (0.13, 1.67)	0.90 (−0.14, 1.93)	0.94 (0.28, 1.60)
Season			
Cool^a^	2.10 (1.47, 2.73)	1.15 (0.05, 2.24)	2.64 (1.87, 3.42)
Warm^b^	−1.10 (−1.67, 0.52)	0.80 (−0.44, 2.03)	0.54 (−0.08, 1.16)

PM_10_, particulate matter with an aerodynamic diameter less than 10 µm; SO_2_, sulfur dioxide; NO_2_, nitrogen dioxide.

^a^Cool season: from November to April.

^b^Warm season: from May to October.

Figure graphically shows that the concentration-response relationships for PM_10_ and NO_2_ with ED visits for CHD were almost linearly positive, without any thresholds; however, the curve for SO_2_ was almost flat, supporting its insignificant association with CHD.

**Figure.**
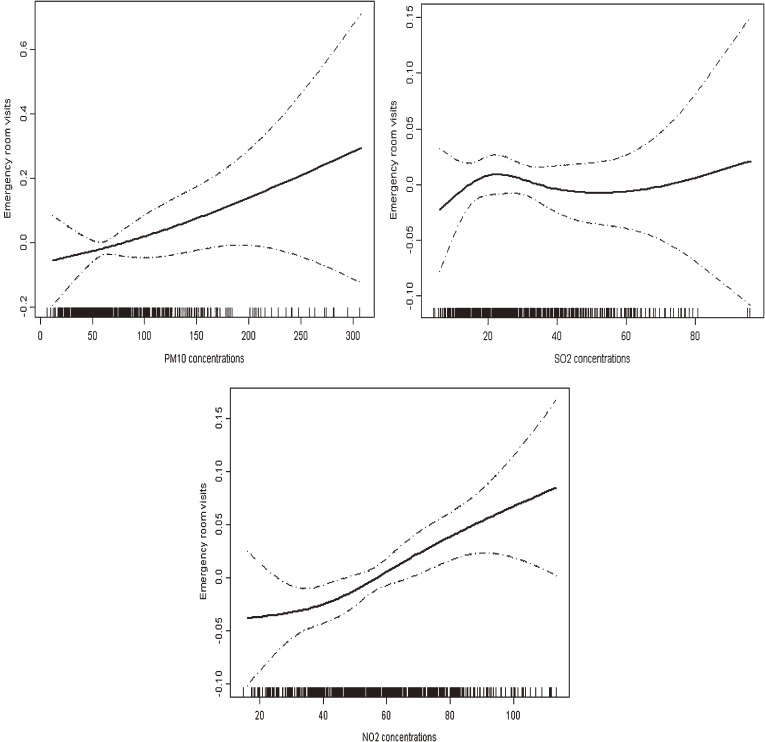
The concentration-response relationships of air pollutant concentrations and emergency room visits for coronary heart disease in Shanghai, China, from 2010–2012. The X-axis is the pollutant concentrations (µg/m^3^) at the concurrent day. The Y-axis is the log-relative risk on the outpatient visits for coronary heart disease. The solid lines indicate the estimated mean change in the log-relative risk, and the dotted lines represent the 95% confidence intervals of the estimates. PM_10_, particulate matter with an aerodynamic diameter less than 10 µm; SO_2_, Sulfur dioxide; NO_2_, Nitrogen dioxide.

We performed several additional analyses to check the sensibility of our main results. We also ran two-pollutant models after adding other pollutants to the models in turn. Table 4 shows that the estimated associations of PM_10_ with CHD decreased moderately after adjusting for SO_2_ and NO_2_ but remained statistically significant. The effect of SO_2_ decreased substantially after controlling for PM_10_ and NO_2_, while the effect of NO_2_ changed little in response to the adjustment of PM_10_ and SO_2_. Using alternative df per year, we found that the results changed substantially. For another sensitivity analysis, the effect became null after replacing CHD with injuries, suggesting that our results might be not attributable to chance of modeling. Finally, the acute effects of air pollution on five subtypes of CHD did change substantially using an alternative time-stratified case-crossover design (data not shown).

**Table 4.** Percent increase (mean and 95% confidence intervals) in emergency room visits for coronary heart disease associated with a 10-µg/m^3^ increase in pollutant concentrations on the concurrent day using single- and two-pollutant models in Shanghai, China, from 2010–2012

	Model	Effect estimates
PM_10_	Without adjustment	1.10 (0.33, 1.87)
	Adjusting for SO_2_	0.90 (0.11, 1.69)
	Adjusting for NO_2_	0.71 (0.08, 1.34)
SO_2_	Without adjustment	0.90 (−0.14, 1.93)
	Adjusting for NO_2_	0.30 (−0.94, 1.53)
	Adjusting for PM_10_	−0.40 (−1.71, 0.92)
NO_2_	Without adjustment	1.44 (0.63, 2.26)
	Adjusting for SO_2_	1.34 (0.47, 2.22)
	Adjusting for PM_10_	1.49 (0.70, 2.29)

PM_10_, particulate matter with an aerodynamic diameter less than 10 µm; SO_2_, sulfur dioxide; NO_2_, nitrogen dioxide.

## DISCUSSION

Although there is ample evidence that short-term exposure to ambient air pollution contributes to acute cardiac morbidity and mortality, knowledge is scarce with regard to effects on the subtypes of CHD. Consistent with previous studies, this study supported a significant effect of air pollution on the acute exacerbation of CHD, and further suggested that this effect may vary greatly with different clinical types of CHD. To our knowledge, the present study is one of the few studies worldwide to explore the effects of air pollution on specific types of CHD. Our results also provide scientific evidence that outdoor air pollution is associated with increased risk of ED visits for CHD subtypes in China.

Few prior studies have simultaneously examined and compared the effects of air pollution on various clinical types of CHD. We found that the association of air pollution and CHD varied greatly by clinical type, with strongest effects on SCD, moderate effects on angina and AMI, weak effects on ICM, and no effect on occult CHD. Epidemiological studies have consistently reported the significant associations of air pollutants on CHD as a whole and two subtypes (SCD and AMI).^18^^,^^19^ SCD is the most severe manifestation of CHD, constituting almost half of cardiac deaths in developed countries.^20^

In the current study, we found that air pollution had the most significant and strongest effects on SCD among CHD subtypes. SCD has been consistently associated with outdoor air pollution in several studies in Italy, Denmark, the US, and Australia.^18^^,^^21^ Evidence was also abundant concerning the association of air pollution with AMI, another severe manifestation of CHD. Mustafic et al performed a systematic review and meta-analysis of the association between air pollution and AMI.^22^ In their examination of 34 studies, they found that PM_10_, PM_2.5_, SO_2_, NO_2_, and carbon monoxide (but not ozone) were significantly associated with a near-term increase in AMI risk. Our estimates were mostly consistent with this meta-analysis, but the magnitude was slightly larger. Previous evidence was very scarce with regard to the possible effects of air pollution on other CHD types, although these types are more clinically common than SCD and AMI. We found the effect estimates were moderate for angina, weak for ICM, and insignificant for occult CHD. The varying effects of air pollution on subtypes of CHD might reflect the different susceptibilities of CHD subtypes to the hazardous effects of air pollution, and thus subsequent public health significance should be noted.

The strongest effects of air pollution generally occurred on the concurrent day and the previous day, and longer lag days did not produce significant effects. This was mostly consistent with other studies linking air pollution and acute cardiovascular events.^18^^,^^23^ The notion that air pollution can trigger cardiovascular events has been supported by several possible mechanisms involving the induction of inflammation and oxidative stress, endothelial vasoconstriction of the coronary arteries, abnormal regulation of the cardiac autonomic system, increased blood viscosity, and vasospasm. All these pathways can lead to plaque rupture, ischemia, and arrhythmia in CHD patients.^5^^,^^22^ However, the underlying mechanisms for the varying effects in air pollution on different manifestations of CHD remains unclear. Further studies are still needed to understand the potentially different pathways of air pollution on CHD subtypes in order to better inform public health policy formation.

Understanding the modifiers of the association between air pollution and CHD is important to the primary prevention of CHD. We found a stronger association in people ≥65 years than in younger people. Aging is a well-known risk factor for most human diseases, so it is plausible that older people are more susceptible to air pollution. We did not detect apparent differences between males and females, although the results showed that males had slightly higher risk. The sex difference was not easily explained because male sex may contribute to an increased risk for cardiovascular events, but a relatively frail physique, more deposition in the lung, higher airway responsiveness, and unfavorable socioeconomic status of females could also make females more susceptible to air pollution.^24^^,^^25^ We found that the air pollution-CHD associations varied greatly by season. Air pollution posed much higher excess risk in the cool period, possibly because of the varying air pollution mixtures and levels, as well as population exposure patterns, in different seasons.^24^ Therefore, CHD patients should be encouraged to avoid exposure to outdoor air pollution, especially in cool seasons.

Air pollution is a complex mixture. In this study, we found that PM_10_ and NO_2_ (but not SO_2_) were strongly and robustly associated with CHD. In China, PM_10_ and NO_2_ typically originate from fossil fuel combustion. Therefore, these two pollutants should be given priority in terms of both air-pollution control and public-health investigations. However, pollutants coexist in the air and correlate with each other, and thus it is not easy for observational epidemiological studies to disentangle the health effects of different pollutants, especially in two-pollutant models.^26^

Understanding the shapes of concentration-response relationships is important for public health assessment and prevention. In this study, we found nearly linear positive curves for PM_10_/NO_2_ and CHD, without any observable thresholds. The curves illustrate that the risks of the exacerbation of CHD could increase linearly corresponding to short-term increases in air pollution levels, and that further alleviation of air pollution could bring appreciable benefits in cardiac health.

Our study has three strengths. First, our sample size of 47 523 ED visits for CHD was quite large, and there were no missing data on air quality during the study period, allowing for sufficient statistical power to detect a significant association. Second, the study is one of the few studies to report the hazardous effects of air pollution on CHD in China. Third, our study is also the first to simultaneously examine and compare the acute effects of air pollution on five clinical subtypes of CHD.

Nevertheless, our work still has several limitations. First, as in most previous time-series studies, we relied on routine measurements from a fixed-site monitoring station as the proxy for the population exposure level, which may result in inevitable measurement error because the ambient monitoring results may differ from the level of personal exposure to air pollutants, as people typically spent most of their time indoors.^27^^,^^28^ To attenuate this error, we used the air quality data derived from a central monitoring station near the hospital and restricted our sample to patients residing within the district. Further, this kind of measurement error has been shown to bias estimates downward.^29^^,^^30^ Second, limited by the data availability, we only collected CHD data from one hospital, decreasing the generalizability of our results; therefore, large-scale studies, especially multi-center studies, are needed to confirm our results. Third, we did not evaluate the effects of fine particles (PM_2.5_) and ozone, because they were not routinely monitored in China during our study period. Fourth, although we did our utmost to identify the different subtypes of CHD, potential misclassification of diagnoses might still exist, especially when patients suffered from several subtypes concurrently.

In summary, our study supports the association between increases in urban background air pollution levels and increased number of ED visits for CHD in Shanghai, China. Further, we found that these risks varied greatly by clinical subtype of CHD, with strongest effects on SCD, moderate effects on AMI and angina, weak effects on ICM, and no effect on occult CHD. To our knowledge, this study is the first to simultaneously report the acute effects of air pollution on five different clinical subtypes of CHD. Our results might be useful for the primary prevention of various types of CHD exacerbated by air pollution.

## ONLINE ONLY MATERIALS

eFigure 1. The scatter plots for daily emergency department visits for coronary heart disease in Shanghai, China, from 2010–2012. The black line represents the smoothed trend using a natural spline with 3 degrees of freedom per year.

eFigure 2. The scatter plots for daily PM_10_ concentrations in Shanghai, China, from 2010–2012. PM_10_, particulate matter with an aerodynamic diameter less than 10 µm. The black line represents the smoothed trend using a natural spline with 3 degrees of freedom per year.

eFigure 3. The scatter plots for daily SO_2_ concentrations in Shanghai, China, from 2010–2012. SO_2_, sulfur dioxide. The black line represents the smoothed trend using a natural spline with 3 degrees of freedom per year.

eFigure 4. The scatter plots for daily NO_2_ concentrations in Shanghai, China, from 2010–2012. NO_2_, nitrogen dioxide. The black line represents the smoothed trend using a natural spline with 3 degrees of freedom per year.
