# Supplementary material for: Acute Effects of Outdoor Air Pollution on Emergency Department Visits Due to Five Clinical Subtypes of Coronary Heart Diseases in Shanghai, China
Source: J Epidemiol. 2014 Nov 5;24(6):452–9. doi: 10.2188/jea.JE20140044 (PMC4213219; doi:10.2188/jea.JE20140044)
Supplement: eFigure 2. [file je-24-452-s002.pdf]

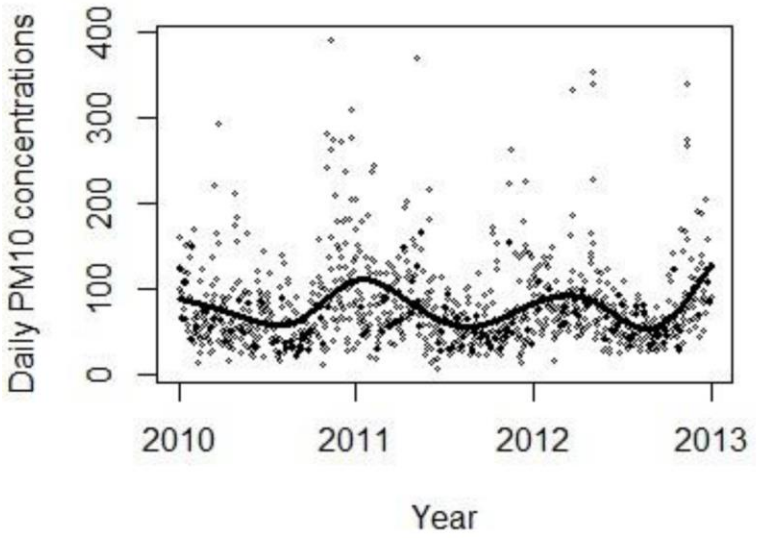

eFigure 2. The scatter plots for daily PM_{10} concentrations in Shanghai, China, from 2010-2012. PM_{10}, particulate matter with an aerodynamic diameter less than 10 μm. The black line represents the smoothed trend using a natural spline with 3 degrees of freedom per year.
